# Sulfadiazine Salicylaldehyde-Based Schiff Bases: Synthesis, Antimicrobial Activity and Cytotoxicity

**DOI:** 10.3390/molecules22091573

**Published:** 2017-09-19

**Authors:** Martin Krátký, Magdaléna Dzurková, Jiří Janoušek, Klára Konečná, František Trejtnar, Jiřina Stolaříková, Jarmila Vinšová

**Affiliations:** 1 Department of Organic and Bioorganic Chemistry, Faculty of Pharmacy in Hradec Králové, Charles University, Akademika Heyrovského 1203, 500 05 Hradec Králové, Czech Republic; vinsova@faf.cuni.cz; 2 Department of Chemistry, Faculty of Science, J. E. Purkinje University, České mládeže 8, 400 96 Ústí nad Labem, Czech Republic; dzurkova.magda@seznam.cz; 3 Department of Pharmacology and Toxicology, Faculty of Pharmacy, Charles University, Akademika Heyrovského 1203, 500 05 Hradec Králové, Czech Republic; janousj2@faf.cuni.cz (J.J.); trejnarf@faf.cuni.cz (F.T.); 4 Department of Biological and Medical Sciences, Faculty of Pharmacy, Charles University, Akademika Heyrovského 1203, 500 05 Hradec Králové, Czech Republic; konecna@faf.cuni.cz; 5 Laboratory for Mycobacterial Diagnostics and Tuberculosis, Regional Institute of Public Health in Ostrava, Partyzánské námĕstí 7, 702 00 Ostrava, Czech Republic; Jirina.Stolarikova@zu.cz

**Keywords:** antibacterial activity, antifungal activity, antimycobacterial activity, cytotoxicity, Schiff bases, sulfadiazine, sulfonamides

## Abstract

The resistance among microbes has brought an urgent need for new drugs. Thus, we synthesized a series of Schiff bases derived from the sulfa drug sulfadiazine and various salicylaldehydes. The resulting 4-[(2-hydroxybenzylidene)amino]-*N*-(pyrimidin-2-yl)benzene-sulfonamides were characterized and evaluated against Gram-positive and Gram-negative bacteria, yeasts, moulds, *Mycobacterium tuberculosis*, nontuberculous mycobacteria (*M. kansasii*, *M. avium*) and their cytotoxicity was determined. Among bacteria, the genus *Staphylococcus*, including methicillin-resistant *S. aureus*, showed the highest susceptibility, with minimum inhibitory concentration values from 7.81 µM. The growth of *Candida* sp. and *Trichophyton interdigitale* was inhibited at concentrations starting from 1.95 µM. 4-[(2,5-Dihydroxybenzylidene)amino]-N-(pyrimidin-2-yl)-benzenesulfonamide was identified as the most selective Schiff base for these strains with no apparent cytotoxicity and a selectivity index higher than 16. With respect to *M. tuberculosis* and *M. kansasii* that were inhibited within the range of 8 to 250 µM, unsubstituted 4-[(2-hydroxy-benzylidene)amino]-*N*-(pyrimidin-2-yl)benzenesulfonamide meets the selectivity requirement. In general, dihalogenation of the salicylic moiety improved the antibacterial and antifungal activity but also increased the cytotoxicity, especially with an increasing atomic mass. Some derivatives offer more advantageous properties than the parent sulfadiazine, thus constituting promising hits for further antimicrobial drug development.

## 1. Introduction

Schiff bases are condensation products of primary (aromatic) amines with aldehydes or ketones carrying the azomethine (imino) moiety (-CR=N-). They are considered versatile pharmacophores for various pharmacological activities where the azomethine group has been demonstrated to be critical to the bioactivity. For example, Schiff bases, whether of natural or non-natural origin, have exhibited promising antibacterial, antitubercular, antifungal, antiparasitic, antiviral, antioxidant, anticancer, analgesic, anti-inflammatory properties etc. [1,2,3]. Altogether, they represent very frequently used and useful scaffold in medicinal chemistry.

**Figure 1:**
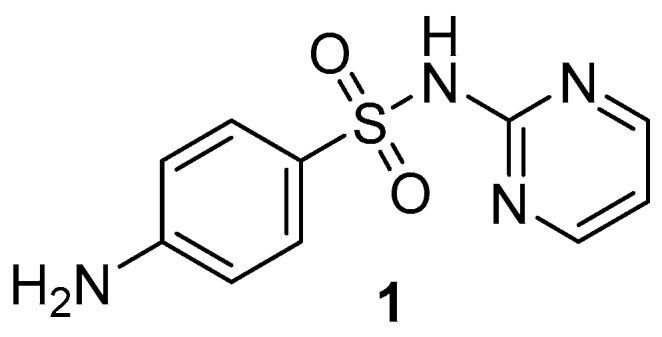
Sulfadiazine **1**.

**Figure 2:**
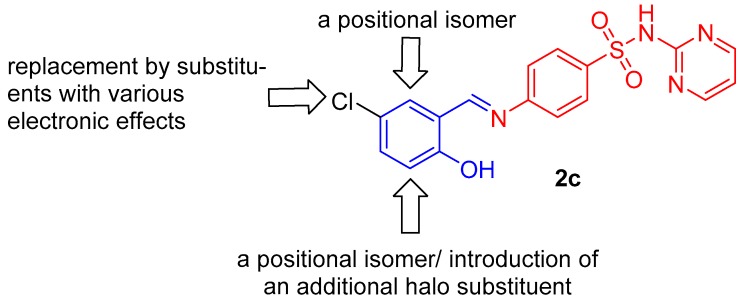
Rational design of novel Schiff bases combining sulfadiazine (red) and salicylaldehyde (blue) scaffolds based on structural modifications of **2c**.

Schiff bases of substituted salicylaldehydes (2-hydroxybenzaldehydes) are well-known antimicrobial agents in “free” form or as ligands in metallic complexes [4,5,6,7]. Similar biological action have been reported for Schiff bases of various sulfonamides [4,8,9]. In addition, cotrimoxazole, sulfamethoxazole and sulfadiazine (**1**, SDZ, Figure 1) have exhibited activity against *Mycobacterium tuberculosis* [10] as well as nontuberculous (atypical) mycobacteria (NTM) [11] at clinically achievable concentrations after administration *per os*. To the best of our knowledge, there is no report about an identical property of sulfathiazole, used now only topically, or other sulfonamides. El-Baradie reported [12] an antibacterial activity of a SDZ-based Schiff base with unsubstituted salicylaldehyde. It exhibited minimum inhibitory concentrations (MICs) of 100–250 µg/mL for both Gram-positive and Gram-negative bacteria. The Schiff base derived from 5-bromosalicylaldehyde exhibited a broad spectrum of antibacterial and antifungal properties [13]. Isosteric 4-(5-chloro-2-hydroxybenzylideneamino)-*N*-(pyrimidin-2-yl)benzenesulfonamide (**2c**, Figure 2) showed activity against *Staphylococcus aureus*, including a methicillin-resistant strain (MRSA), *M. tuberculosis* and *Mycobacterium avium* (MIC values within the range of 125–250 µM) whereas it caused a significantly higher inhibition of *Mycobacterium kansasii* strains (8–32 µM) [4].

Keeping in mind these facts, we decided to combine two well-established pharmacophores for antimicrobial activity, sulfadiazine and salicylaldehyde, into one molecular Schiff base entity (Figure 2) and to screen systematically their antimicrobial and cytotoxic properties. In our study [4], the activities of sulfadiazine amides and imines based on the salicylic scaffold were comparable against the majority of mycobacterial strains and drug-susceptible *Staphylococcus aureus*, but further research discovered that the amides are significantly more toxic and less selective for eukaryotic cells. This research is justified also by an increasing resistance of microbes to current antimicrobial drugs.

Based on previous reports about 5-chlorosalicyladehyde-based Schiff bases [4,6], we designed compounds **2a** without any substitution of the salicylic scaffold, with chlorine replaced by another halogen (5-F, Br, I; negative inductive, −I, and positive mesomeric, +M, effects), other functional groups with different electronic properties: electron donating groups—alkyls (CH_3_, *tert*-butyl), OH and CH_3_O as well as NO_2_ as an electron withdrawing group. Additionally, based on our previous report [4], we explored positional isomers of 4-[(5-chloro-2-hydroxybenzylidene)amino]-*N*-(pyrimidin-2-yl)-benzenesulfonamide (**2c**, 3-Cl, 6-Cl) and 3,5-dihalogen derivatives (3,5-Cl_2_, 3,5-I_2_, 3-Br-5-Cl and 3-I-5-Cl). The drug design based on rational modifications of **2c** is depicted in Figure 2. We also synthesized the Schiff base of 5-nitrofuran-2-carbaldehyde (5-nitrofurfural; **2q**), a known pharmacophore for antimicrobial activity (e.g., [14,15]). This report is focused on chemical synthesis, characterization and initial biological evaluation (antimicrobial screening, toxicity for eukaryotic cells) to identify the most promising candidates for advanced microbiological and pharmacological tests.

## 2. Results and Discussion

### 2.1. Chemistry

**Scheme 1:**
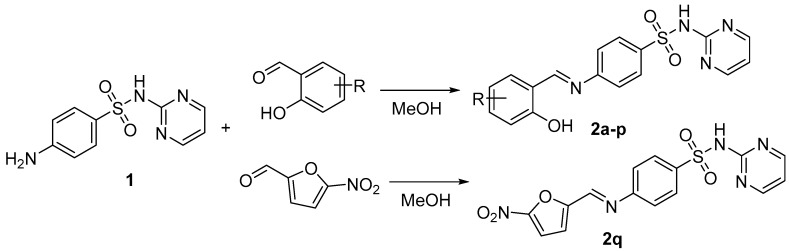
Synthesis of sulfadiazine Schiff bases **2** (MeOH = methanol; R = H, 5-F, 5-Cl, 5-Br, 5-I, 5-NO_2_, 5-Me, 5-MeO, 5-OH, 5-*tert*-Bu, 3-Cl, 6-Cl, 3,5-Cl_2_, 3-Br-5-Cl, 3-I-5-Cl, 3,5-I_2_).

Sulfadiazine-derived Schiff bases **2** were prepared by reactions of SDZ **1** and the corresponding aldehydes (salicylaldehydes, 5-nitrofuran-2-carbaldehyde) in boiling methanol (MeOH) for 5 h (Scheme 1). Aldehydes were added to a hot methanolic suspension of SDZ. The poor solubility of SDZ in MeOH is a reason for an extended reaction time in contrast to previous work [4]. The yields were good, within the range of 85–95%, with 4-[(2-hydroxy-5-nitrobenzylidene)amino]-*N*-(pyrimidin-2-yl)benzenesulfonamide (**2f**) being an exception (74 %). In this case, it was beneficial to dissolve 5-nitrosalicylaldehyde in hot MeOH and then add the sulfonamide; the reaction time was extended to 8 h.

All of the compounds (Table 1) were characterized by ^1^H-, ^13^C-NMR, IR spectra and melting points; their purity was checked additionally by elemental analysis. In the ^1^H-NMR spectra of Schiff bases **2**, an azomethine (CH=N) singlet appeared at 9.16–8.89 ppm. The phenolic hydrogen was observed in a comparatively broader range of 13.93–11.68 ppm. IR spectroscopy displayed a typical sharp and strong azomethine (-CH=N-) band observed at 1582–1570 cm^−1^.

### 2.2. Antimicrobial Activity

The sulfadiazine derivatives **1**–**2** were screened in vitro for their antimicrobial properties. The panel of pathogens involved *Staphylococcus aureus* CCM 4516/08, methicillin-resistant *Staphylococcus aureus* H 5996/08 (MRSA), *Staphylococcus epidermidis* H 6966/08, *Enterococcus faecalis* J 14365/08 (Gram-positive bacteria), *Escherichia coli* CCM 4517, *Klebsiella pneumoniae* D 11750/08, extended spectrum beta-lactamase (ESBL)-positive *Klebsiella pneumoniae* J 14368/08, and *Pseudomonas aeruginosa* CCM 1961 (Gram-negative strains) (Table 1), mycobacteria *Mycobacterium tuberculosis* 331/88 (H_37_Rv), *Mycobacterium avium* 330/88, *Mycobacterium kansasii* 235/80 and the clinical isolate 6509/96 (Table 2) [16], and fungal species of *Candida albicans* ATCC 44859, *Candida tropicalis* 156, *Candida krusei* E28, *Candida glabrata* 20/I, *Trichosporon asahii* 1188, *Aspergillus fumigatus* 231, *Lichtheimia corymbifera* 272, and *Trichophyton interdigitale* 445 (Table 3) [17]. This panel of twenty microbial species covers a wide range of important human pathogens including those with an acquired resistance. It is a useful screening tool for an initial identification of potential antimicrobial activity of novel compounds.

Bacitracin (BAC), fluconazole (FLU) and isoniazid (INH) were employed as the comparative drugs for antibacterial, antifungal and antimycobacterial activity, respectively. The parent sulfadiazine **1** showed only a weak action against both strains of *Staphylococcus aureus* (Table 1), and no antifungal properties, but it is an antimycobacterial agent (Table 2). Four Schiff bases (compounds **2b**, **2j**–**l**) showed no significant antibacterial activity (Table 1). In general, the action against Gram-negative species is only negligible (MICs starting from 125 µM). 4-[(2-Hydroxy-3,5-diiodobenzylidene)amino]-*N*-(pyrimidin-2-yl)benzenesulfonamide (**2p**) showed the lowest MIC values, followed by other halogenated Schiff bases **2d**, **2e**, **2o**. Among Gram-positive pathogens, *Enterococcus* was inhibited by six derivatives (compounds **2a**, **2e**, **2f**, **2i**, **2o**, **2p**) with 4-[(2-hydroxybenzylidene)amino]-*N*-(pyrimidin-2-yl)benzenesulfonamide (**2a**) showing superiority (31.25/62.5 µM). Thus, the antibacterial properties are manifested especially for *Staphylococci*. MICs tend to be even lower for MRSA when compared to the methicillin-susceptible strain (e.g., **2g**). For *S. aureus*, the 3,5-dihalogen substitution is translated into an enhanced activity (MICs of 7.81–62.5 µM) where heavier atoms contribute to more intense action (7.81–62.5 µM). Another substituent decreasing MIC values is a second hydroxyl group (i.e., **2i**) in the *para*-position with respect to the salicylic hydroxyl with a uniform MIC of 31.25 µM.

The rest of sulfadiazines **2** share MICs of 125–500 µM. The analysis of the effect of the chlorine position on the salicylic ring revealed that the activity is conferred by its presence at the position 5 while its shift to another carbon hampers antibacterial activity completely (**2c** vs. **2k**–**l**). 3,5-Diiodosalicylidene sulfadiazine **2p** was also superior for the inhibition of *S. epidermidis* (7.81 µM), followed by 5-OH (**2i**, 15.62 µM), other 3,5-dihalogen (**2m**–**o**) and 5-nitrofuran **2q** (31.25 µM) derivatives. The diiodo derivative **2p** was superior to bacitracin against *S. epidermidis*, and five compounds (**2i**, **2m**–**o**, **2q**) were fully comparable. The activities of BAC and several Schiff bases (compounds **2n**–**p**) are identical against *S. aureus*. Considering *Enterococcus* sp., the derivative **2a** exhibited comparable MIC values.

All of the compounds **2** were able to inhibit both *M. tuberculosis* and NTM in vitro with MIC values within the range of 8–1000 µM. Both strains of *M. kansasii* showed the highest rate of susceptibility (MIC ≥ 8 µM), followed by *M. tuberculosis* (≥16 µM) and *M. avium* (MIC values of 125–500 µM). The best results were obtained for the simplest derivative, 4-[(2-hydroxybenzylidene)amino]-*N*-(pyrimidin-2-yl)benzene-sulfonamide (**2a**) with MICs of 8–250 µM but for *M. tuberculosis* and both *M. kansasii* strains its MICs were ≤32 µM. A previously reported derivative of 5-chlorosalicylaldehyde [4] was efficient in vitro against NTM. Although 5-nitrofuran-2-yl is a well-established scaffold in drug design of potential anti-TB drugs [18], the 5-nitrofurylidene derivative **2q** did not meet our expectation of excellent antimycobacterial action (MIC values ≥ 62.5 µM).

Structure-activity relationships for the substitution of the salicylidene fragment are reported in Table 4. In general, the introduction of any substituent does not offer any benefit when compared to unsubstituted Schiff base **2a**. Among single halogens, bromine (compound **2d**) is favourable, in the case of NTM strains concomitantly with 5- and 3-chloro isomers (compounds **2c**, **2l**). 5-Methoxy (compound **2h**) and *tert*-butyl (compound **2j**) groups contribute to the improved action, too. On the other hand, halo 3,5-disubstitution (compounds **2m**–**p**), and a nitro group (compound **2f**) are detrimental for the inhibition of all mycobacteria. For NTM, a fluorine atom (compound **2b**), 5-methyl (compound **2g**), or 5-nitrofurylidene (compound **2q**) abrogate the activity. Although lipophilicity represents an important factor influencing antimycobacterial action, no direct correlation between lipophilicity and MIC values was identified.

Drawing a comparison to INH, none of the Schiff bases **2** exceeded its activity against *M. tuberculosis*. For the clinical isolate *M. kansasii* 6509/96, five compounds (**2a**, **2c**, **2h**, **2j**, and **2l**) were comparable after 7 and/or 14 d of incubation (± one dilution). In contrast, eleven derivatives (**2a**, **2c**–**e**, **2h**, **2j**–**n**, **2p**) inhibited the growth of *M. avium* at the concentration of 250 µM that is tolerated in the case of INH. Similarly, all of the derivatives **2** surpassed INH against *M. kansasii* 235/80. 

Unfortunately, the modification of the parent sulfadiazine **1** to the derivatives **2** did not bring any significant improvement of antimycobacterial properties but the most active Schiff bases (**2a**, **2d**, **2h**, **2j**, and **2l**) exhibited activity comparable to that of **1** (± one dilution).

Three Schiff bases (**2b**, **2g**, **2k**) lacked antifungal activity (Table 3), two others (**2a**, **2j**) inhibited only *T. interdigitale* at 125 and 62.5 µM, respectively. Three derivatives showed MIC values against several strains starting from 250 µM (**2f**, **2h**, **2q**) with *T. interdigitale* being an exception (MIC of 62.5 µM for **2f** and **2h**). In general, this strain showed the highest susceptibility from all of the investigated fungi with MIC values of 1.95–250 µM. 3-I-5-Cl (**2o**), 3,5-I_2_ (**2p**), 5-OH (**2j**) were found as preferred substitution patterns for salicylic ring that are superior to FLU. Additional Schiff bases (**2d**–**f**, **2h**, **2j**, **2l**, and **2n**) produced lower MICs after 120 h of incubation. On the other hand, *L. corymbifera* is at least susceptible strain inhibited only by six derivatives (**2f**, **2i**, **2m**–**p**) with MICs ≥ 62.5 µM.

Nine compounds **2d**, **2e**, **2i**, and **2l**–**q** uniformly affected yeasts (*Candida* sp., *T. asahii*), three 5-substituted salicylidenesulfadiazines (5-Cl **2c**, 5-NO_2_ **2f**, and 5-CH_3_O **2h**) were effective in vitro against only some strains. Similar to the antibacterial activity, dihalogen derivatives containing iodine (**2o**–**p**, MICs of 3.9–7.81 µM) or the second phenolic group (**2i**, 15.62–31.25 µM) showed the highest in vitro potency, which was significantly superior to that of the fluconazole; *C. albicans* represented an exception. Satisfactory results are also conferred by 3,5-dichloro- and 3-bromo-5-iodo-salicylaldehyde scaffolds (compounds **2m**, **2n,** 31.25–125 µM), followed by a single halogen (Cl, Br, I) substitution pattern and it correlates positively with an increasing atomic mass and consequent lipophilicity. The comparison of isomeric monochlorinated Schiff bases (**2c**, **2k**, **2l**) revealed the order: 3-Cl (**2l**) > 5-Cl (**2k**) > 6-Cl (inactive **2k**).

Seeking insights into the potential mechanism of action, we evaluated the inhibition potency of selected salicylidenesulfonamides against isocitrate lyase, but they were virtually inactive in spite of the substitution pattern (i.e., their inhibition rates were lower than 10% at the single concentration of 100 µM).

### 2.3. Cytotoxicity and Selectivity

The cytotoxicity of the investigated compounds **1** and **2** was measured using the standard hepatic cell line HepG2 (Table 5) [16]. The used CellTiter 96 assay is based on the reduction of tetrazolium dye in living cells to formazan. This reduction is related to availability of NADH or NADPH. The parameter IC_50_, i.e., the concentration that reduces the viability of the cells to 50% of the maximal viability, was used as a measure of cytotoxicity. It was not possible to determine the exact IC_50_ values of two derivatives (compounds **2a**, **2b**) due to their limited solubility in the cell culture medium used in this experiment.

We can divide the investigated Schiff bases **2** into three groups according to their cytotoxicity for HepG2 cells. The first one covers comparatively non-toxic compounds **1**, **2a**, **2b**, and **2g**–**i** (IC_50_ values > 250 µM). The group of the compounds with an intermediate toxicity and IC_50_s in the range of 102.2 to 160.5 µM consists of **2d**–**f**, and **2k**–**m**. The last group includes sulfonamides with an increased cytotoxicity (IC_50_ ≤ 100 µM: **2j**, **2n**–**q**). 4-{[(5-Nitrofuran-2-yl)methylene]amino}-*N*-(pyrimidin-2-yl)benzene-sulfonamide (**2q**) was found to be the most toxic substance (IC_50_ = 16.0 µM). Obviously, the replacement of the salicyl ring by 5-nitrofuran-2-yl produced a more pronounced toxicity for mammalian cells. The majority of the modifications of the parent sulfadiazine **1** (IC_50_ > 1500 µM) increased the cytotoxicity by up to two orders of magnitude for the most toxic compounds. Generally, the enhanced cytotoxicity is observed in the presence of 3,5-halogen disubstitution and a *tert*-butyl moiety. Among particular halogens, the cytotoxicity correlates positively with an increased atomic mass. The presence of a non-bulky electron-donating group (CH_3_, CH_3_O, OH) or hydrogen results in less cytotoxic compounds (i.e., **2a**, **2g**–**i**).

We calculated the selectivity indices (SIs) for *M. tuberculosis*, both strains of *M. kansasii*, MRSA, *S. epidermidis*, all of the *Candida* species and *T. interdigitale* (Table 5). The SI is defined as the ratio of IC_50_ to MIC, and values higher than 10 indicate rather acceptable toxicity (based on the analogy of the therapeutic index). Considering mycobacteria, in addition to the parent sulfadiazine **1**, two compounds exhibited targeted selectivity: Unsubstituted salicylidene sulfadiazine **2a** (SI > 15.63) followed by 5-methoxy derivative **2h** after 14 d of the incubation. 3-Chlorosalicylaldehyde-based Schiff base **2l** provided for *M. kansasii* a borderline SI value after 7 and 14 d. 4-[(2,5-Dihydroxybenzylidene)amino]-*N*-(pyrimidin-2-yl)benzenesulfonamide (**2i**) showed the best safety profile for *Staphylococci* and fungi (>16), especially for the filamentous fungus *T. interdigitale* (>256.41). For this strain, the dihalogenated derivative **2o** was selective too (SI = 30.37).

## 3. Materials and Methods

### 3.1. Chemistry

#### 3.1.1. General Information

All of the reagents and solvents were purchased from Sigma-Aldrich (St. Louis, MO, USA) or Penta Chemicals (Prague, Czech Republic) and were used as received. The reactions and the purity of the products were monitored by thin-layer chromatography (TLC) with a mixture of dichloromethane with *n*-hexane and methanol (4:3:1, *v/v*) as the eluent. The plates were coated with 0.2 mm Merck 60 F254 silica gel (Merck Millipore, Darmstadt, Germany) and were visualised by UV irradiation (254 nm). The melting points were determined using a B-540 melting point apparatus (Büchi, Flawil, Switzerland) with open capillaries, and the reported values are uncorrected. The elemental analysis (C, H, N) was performed with an automatic microanalyser instrument CHNS-O CE (Fisons EA 1110, Milano, Italy). The infrared spectra (ATR) were recorded on a Nicolet 6700 FT-IR spectrometer (ThermoFisher Scientific, Waltham, MA, USA) in the range of 600 to 4000 cm^−1^. The NMR spectra were measured in DMSO-*d_6_* at ambient temperature using a Varian VNMR S500 instrument (500 MHz for ^1^H and 125 MHz for ^13^C; Varian Inc., Palo Alto, CA, USA). The chemical shifts, δ, are given in ppm with respect to tetramethylsilane as the internal standard. The coupling constants (*J*) are reported in Hz. The calculated log*P* values (Clog*P*), which are the logarithms of the partition coefficients for octan-1-ol/water, were determined using the CS ChemOffice Ultra version 16.0 program (CambridgeSoft, Cambridge, MA, USA).

#### 3.1.2. Synthesis

**Figure 3:**
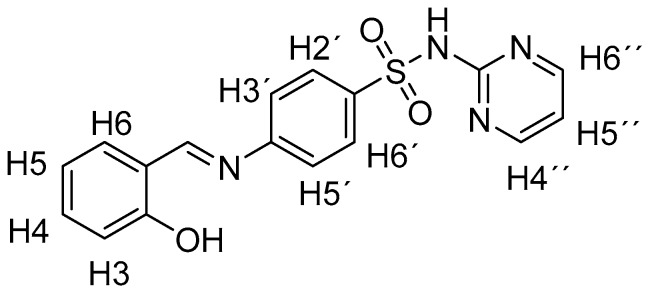
Numbering of H atoms in NMR spectra.

Sulfadiazine **1** (1 mmol, 250.3 mg) was suspended in methanol (MeOH, 10 mL), and 1.1 mmol of the appropriate aldehyde was added in one portion under vigorous stirring. The solution was refluxed for 5 h and then stirred at room temperature overnight. The resulting crystals were filtered off, washed with a small amount of MeOH and then acetonitrile, and dried. The crystals were recrystallized from MeOH or tetrahydrofuran/*n*-hexane mixture if necessary. The identity of known compounds (i.e., **2a**, **2c**, **2d**, and **2m**) was confirmed by NMR (^1^H and ^13^C) and IR spectroscopy. All spectroscopic characteristics were in accordance with previously reported data. The purity was checked additionally by melting points measurement and elemental analysis. The numbering of hydrogen atoms in the ^1^H-NMR spectra is depicted in Figure 3.

*4-[(2-Hydroxybenzylidene)amino]-N-(pyrimidin-2-yl)benzenesulfonamide* (**2a**) [12,19]. Yellow solid; yield 94%; m.p. 246–248.5 °C (lit. [19] 244–245 °C decomp.). IR: 3033, 2937, 2870, 2812, 2740, 1621, 1581 (-HC=N-), 1493, 1446, 1413, 1382, 1339, 1279, 1188, 1165, 1151, 1091, 943, 914, 856, 835, 798, 778, 755, 707, 667 cm^−1^. ^1^H-NMR: δ 12.50 (1H, s, OH), 11.86 (1H, s, SO_2_NH), 8.95 (1H, s, CH=N), 8.51 (2H, d, *J* = 4.9 Hz, H4′′, H6′′), 8.06–8.02 (2H, m, H2′, H6′), 7.68 (1H, dd, *J* = 7.7 Hz, *J* = 1.6 Hz, H6), 7.56–7.52 (2H, m, H3′, H5′), 7.44 (1H, dd, *J* = 7.8 Hz, *J* = 1.7 Hz, H4), 7.06 (1H, t, *J* = 4.9 Hz, H5′′), 7.00–6.96 (2H, m, H3, H5). ^13^C-NMR: δ 165.49, 160.41, 158.54, 157.03, 152.40, 138.16, 134.17, 132.72, 129.24, 121.88, 119.53, 119.48, 116.88, 115.98. Anal. Calcd. for C_17_H_14_N_4_O_3_S (354.38): C, 57.62; H, 3.98; N, 15.81. Found: C, 57.71; H, 4.08; N, 15.65.

*4-[(5-Fluoro-2-hydroxybenzylidene)amino]-N-(pyrimidin-2-yl)benzenesulfonamide* (**2b**). Yellowish-orange solid; yield 92%; m.p. 273–276 °C (decomp.). IR: 3032, 2939, 2870, 2812, 2736, 1570 (-HC=N-), 1483, 1444, 1414, 1338, 1269, 1251, 1220, 1163, 1144, 1091, 943, 879, 860, 835, 798, 776, 702, 669 cm^−1^. ^1^H-NMR: δ 12.07 (1H, s, OH), 11.86 (1H, s, SO_2_NH), 8.90 (1H, s, CH=N), 8.51 (2H, d, *J* = 4.8 Hz, H4′′, H6′′), 8.07–8.02 (2H, m, H2′, H6′), 7.55–7.49 (3H, m, H6, H3′, H5′), 7.31 (dd, *J* = 8.8 Hz, *J* = 3.2 Hz, H4), 7.06 (1H, t, *J* = 4.9 Hz, H5′′), 7.01–6.98 (1H, m, H3). ^13^C-NMR: δ 163.68 (d, *J* = 2.5 Hz), 158.54, 157.01, 156.54, 155.18 (d, *J* = 235.1 Hz), 152.55, 138.31, 129.25, 121.84, 121.11 (d, *J* = 23.6 Hz), 119.96 (d, *J* = 7.6 Hz), 118.36 (d, *J* = 7.6 Hz), 116.68 (d, *J* = 23.4 Hz), 112.28. Anal. Calcd. for C_17_H_13_FN_4_O_3_S (372.37): C, 54.83; H, 3.52; N, 15.05. Found: C, 54.90; H, 3.78; N, 14.94.

*4-[(5-Chloro-2-hydroxybenzylidene)amino]-N-(pyrimidin-2-yl)benzenesulfonamide* (**2c**) [4]

*4-[(5-Bromo-2-hydroxybenzylidene)amino]-N-(pyrimidin-2-yl)benzenesulfonamide* (**2d**) [13]. Orange solid; yield 89%; m.p. 273.5–275.5 °C (lit. [13] 246–248 °C). Anal. Calcd. for C_17_H_13_BrN_4_O_3_S (433.28): C, 47.13; H, 3.02; N, 12.93. Found: C, 46.97; H, 3.21; N, 13.04.

*4-[(2-Hydroxy-5-iodobenzylidene)amino]-N-(pyrimidin-2-yl)benzenesulfonamide* (**2e**). Orange solid; yield 92%; m.p. 273–274.5 °C (decomp.). IR: 3038, 2944, 2871, 2812, 2736, 1579 (-HC=N-), 1558, 1493, 1443, 1411, 1340, 1280, 1180, 1163, 1093, 945, 912, 860, 837, 823, 797, 783, 715, 671 cm^−1^. ^1^H-NMR: δ 12.42 (1H, s, OH), 11.86 (1H, s, SO_2_NH), 8.89 (1H, s, CH=N), 8.51 (2H, d, *J* = 4.9 Hz, H4′′, H6′′), 8.06–8.00 (3H, m, H6, H2′, H6′), 7.70 (1H, dd, *J* = 8.7 Hz, *J* = 2.3 Hz, H4), 7.54–7.49 (2H, m, H3′, H5′), 7.05 (1H, t, *J* = 4.9 Hz, H5′′), 6.83 (1H, d, *J* = 8.7 Hz, H3). ^13^C-NMR: δ 163.59, 159.90, 158.53, 157.01, 152.34, 141.93, 139.88, 138.32, 129.24, 122.12, 121.89, 119.64, 112.29, 81.10. Anal. Calcd. for C_17_H_13_IN_4_O_3_S (480.28): C, 42.51; H, 2.73; N, 11.67. Found: C, 42.33; H, 2.60; N, 11.89.

*4-[(2-Hydroxy-5-nitrobenzylidene)amino]-N-(pyrimidin-2-yl)benzenesulfonamide* (**2f**). Yellowish-orange solid; yield 74%; m.p. 277–279 °C (decomp.). IR: 3045, 2733, 1581 (-HC=N-), 1530, 1487, 1444, 1411, 1340, 1338, 1298, 1161, 1093, 946, 840, 808, 785, 753, 733, 673 cm^−1^. ^1^H-NMR: δ 13.51 (1H, s, OH), 11.87 (1H, s, SO_2_NH), 9.10 (1H, s, CH=N), 8.69 (1H, d, *J* = 2.9 Hz, H6), 8.52 (2H, d, *J* = 4.9 Hz, H4′′, H6′′), 8.29 (1H, dd, *J* = 9.1 Hz, *J* = 2.9 Hz, H4), 8.09–8.03 (2H, m, H2′, H6′), 7.60–7.55 (2H, m, H3′, H5′), 7.16 (1H, d, *J* = 9.1 Hz, H3), 7.06 (1H, t, *J* = 4.9 Hz, H5′′). ^13^C-NMR: δ 189.25, 165.92, 163.00, 158.40, 157.0, 139.76, 138.76, 129.98, 129.29, 127.84, 121.96, 119.43, 118.27, 112.30. Anal. Calcd. for C_17_H_13_N_5_O_5_S (399.38): C, 51.13; H, 3.28; N, 17.54. Found: C, 51.00; H, 3.11; N, 17.35.

*4-[(2-Hydroxy-5-methylbenzylidene)amino]-N-(pyrimidin-2-yl)benzenesulfonamide* (**2g**). Orange solid; yield 90%; m.p. 255–258 °C (decomp.). IR: 3587 (O-H), 3035, 2807, 2737, 1576 (-HC=N-), 1491, 1441, 1409, 1378, 1343, 1284, 1263, 1207, 1185, 1157, 1094, 945, 877, 843, 817, 797, 786, 704, 680, 667 cm^−1^. ^1^H-NMR: δ 12.21 (1H, s, OH), 11.82 (1H, s, SO_2_NH), 8.89 (1H, s, CH=N), 8.51 (2H, d, *J* = 4.9 Hz, H4′′, H6′′), 8.06–8.01 (2H, m, H2′, H6′), 7.53–7.49 (2H, m, H3′, H5′), 7.47 (1H, d, *J* = 2.3 Hz, H6), 7.25 (dd, *J* = 8.4 Hz, *J* = 2.1 Hz, H4). 7.05 (1H, t, *J* = 4.9 Hz, H5′′), 6.88 (1H, d, *J* = 8.4 Hz, H3), 2.26 (3H, s, CH_3_). ^13^C-NMR: δ 165.25, 158.39, 158.28, 157.04, 152.58, 138.08, 134.96, 132.32, 129.25, 128.10, 121.83, 119.17, 116.75, 115.98, 20.08. Anal. Calcd. for C_18_H_16_N_4_O_3_S (368.41): C, 58.68; H, 4.38; N, 15.21. Found: C, 58.84; H, 4.22; N, 15.41.

*4-[(2-Hydroxy-5-methoxybenzylidene)amino]-N-(pyrimidin-2-yl)benzenesulfonamide* (**2h**). Orange-yellow solid; yield 86%; m.p. 232–234 °C. IR: 3030, 2734, 1576 (-HC=N-), 1487, 1440, 1410, 1338, 1268, 1215, 1153, 1087, 1035, 939, 876, 868, 852, 831, 820, 797, 754, 716, 694, 668 cm^−1^. ^1^H-NMR: δ 12.25 (1H, s, OH), 11.83 (1H, s, SO_2_NH), 8.91 (1H, s, CH=N), 8.51 (2H, d, *J* = 4.9 Hz, H4′′, H6′′), 8.06–8.01 (2H, m, H2′, H6′), 7.53–7.47 (2H, m, H3′, H5′), 7.27 (1H, d, *J* = 3.1 Hz, H6), 7.08–7.04 (2H, m, H4, H5′′), 6.92 (1H, d, *J* = 9.0 Hz, H3), 3.74 (3H, s, CH_3_). ^13^C-NMR: δ 164.73, 158.54, 157.03, 154.51, 152.77, 152.14, 138.05, 129.25, 121.78, 121.54, 119.44, 117.84, 115.98, 114.80, 55.72. Anal. Calcd. for C_18_H_16_N_4_O_4_S (384.41): C, 56.24; H, 4.20; N, 14.58. Found: C, 56.50; H, 4.31; N, 14.32.

*4-[(2,5-Dihydroxybenzylidene)amino]-N-(pyrimidin-2-yl)benzenesulfonamide* (**2i**). Orange-yellow solid; yield 88%; m.p. 247–249 °C (decomp.). IR: 3491 (O-H), 3032, 2939, 2811, 2730, 1573 (-HC=N-), 1480, 1447, 1412, 1359, 1325, 1280, 1248, 1208, 1182, 1142, 1086, 945, 882, 862, 817, 797, 784, 704, 669 cm^−1^. ^1^H-NMR: δ 11.84 (1H, s, SO_2_NH), 11.68 (1H, s, 2-OH), 9.13 (1H, s, CH=N), 8.84 (1H, s, 5-OH), 8.51 (2H, d, *J* = 4.9 Hz, H4′′, H6′′), 8.06–7.99 (2H, m, H2′, H6′), 7.53–7.45 (2H, m, H3′, H5′), 7.09–7.03 (2H, m, H6, H5′′), 6.89 (1H, dd, *J* = 8.8 Hz, *J* = 3.0 Hz, H4), 6.81 (1H, d, *J* = 8.8 Hz, H3). ^13^C-NMR: δ 164.96, 158.59, 157.04, 153.30, 152.85, 149.92, 137.95, 129.21, 122.13, 121.82, 119.53, 117.55, 116.72, 115.98. Anal. Calcd. for C_17_H_14_N_4_O_4_S (370.38): C, 55.13; H, 3.81; N, 15.13. Found: C, 55.02; H, 3.65; N, 15.37.

*4-{[5-(Tert-butyl)-2-hydroxybenzylidene]amino}-N-(pyrimidin-2-yl)benzenesulfonamide* (**2j**). Orange-yellow solid; yield 90%; m.p. 267.5–270 °C (decomp.). IR: 3034, 2953, 1620, 1576 (-HC=N-), 1489, 1448, 1411, 1333, 1264, 1181, 1152, 1093, 953, 879, 839, 827, 798, 719, 671 cm^−1^. ^1^H-NMR: δ 12.30 (1H, s, OH), 11.83 (1H, s, SO_2_NH), 8.96 (1H, s, CH=N), 8.51 (2H, d, *J* = 4.9 Hz, H4′′, H6′′), 8.06–8.00 (2H, m, H2′, H6′), 7.69 (1H, d, *J* = 2.5 Hz, H6), 7.55–7.51 (2H, m, H3′, H5′), 7.49 (dd, *J* = 8.7 Hz, *J* = 2.5 Hz, H4). 7.06 (1H, t, *J* = 4.9 Hz, H5′′), 6.91 (1H, d, *J* = 8.7 Hz, H3), 1.27 (9H, s, CH_3_). ^13^C-NMR: δ 166.16, 158.86, 158.56, 157.32, 152.85, 141.97, 138.32, 131.83, 129.53, 129.20, 122.12, 119.00, 116.81, 112.58, 34.27, 31.60. Anal. Calcd. for C_21_H_22_N_4_O_3_S (410.49): C, 61.45; H, 5.40; N, 13.65. Found: C, 61.36; H, 5.28; N, 13.49.

*4-[(2-Chloro-6-hydroxybenzylidene)amino]-N-(pyrimidin-2-yl)benzenesulfonamide* (**2k**). Yellow solid; yield 86%; m.p. 248–250 °C (decomp.). IR: 3032, 2943, 2810, 2734, 1580 (-HC=N-), 1489, 1446, 1408, 1339, 1277, 1177, 1158, 1092, 945, 929, 864, 849, 822, 797, 780, 734, 724, 670, 652 cm^−1^. ^1^H-NMR: δ 13.86 (1H, s, OH), 11.90 (1H, s, SO_2_NH), 9.16 (1H, s, CH=N), 8.52 (2H, d, *J* = 4.9 Hz, H4′′, H6′′), 8.09–8.04 (2H, m, H2′, H6′), 7.65–7.60 (2H, m, H3′, H5′), 7.45 (1H, t, *J* = 8.2 Hz, H4), 7.10–7.04 (2H, m, H5, H5′′), 6.98 (1H, d, *J* = 8.4 Hz, H3). ^13^C-NMR: δ 162.72, 162.55, 158.54, 156.98, 151.00, 138.91, 135.90, 135.11, 129.29, 122.19, 120.50, 116.82, 115.99, 115.64. Anal. Calcd. for C_17_H_13_ClN_4_O_3_S (388.83): C, 52.51; H, 3.37; N, 14.41. Found: C, 52.39; H, 3.39; N, 14.21.

*4-[(3-Chloro-2-hydroxybenzylidene)amino]-N-(pyrimidin-2-yl)benzenesulfonamide* (**2l**). Orange solid; yield 90%; m.p. 265–266.5 °C. IR: 3223, 3041, 2869, 1618, 1576 (-HC=N-), 1489, 1441, 1409, 1360, 1338, 1281, 1202, 1182, 1151, 1115, 1091, 932, 845, 808, 792, 770, 741, 667 cm^−1^. ^1^H-NMR: δ 13.77 (1H, s, OH), 11.90 (1H, s, SO_2_NH), 9.05 (1H, s, CH=N), 8.52 (2H, d, *J* = 4.9 Hz, H4′′, H6′′), 8.09–8.04 (2H, m, H2′, H6′), 7.66–7.60 (4H, m, H4, H6, H3′, H5′), 7.06 (1H, t, *J* = 4.9 Hz, H5′′), 7.01 (1H, t, *J* = 7.8 Hz, H5). ^13^C-NMR: δ 165.91, 158.55, 156.99, 156.52, 150.86, 138.78, 133.91, 132.28, 129.25, 122.10, 120.55, 120.18, 119.96, 115.97. Anal. Calcd. for C_17_H_13_ClN_4_O_3_S (388.83): C, 52.51; H, 3.37; N, 14.41. Found: C, 52.60; H, 3.21; N, 14.61.

*4-[(3,5-Dichloro-2-hydroxybenzylidene)amino]-N-(pyrimidin-2-yl)benzenesulfonamide* (**2m**) [20]. Orange solid; yield 86%; m.p. 274–276 °C (decomp.; lit. [20] 260 °C from acetone). IR: 3036, 2722, 1582 (-HC=N-), 1494, 1453, 1441, 1410, 1337, 1265, 1199, 1182, 1153, 1092, 947, 890, 869, 843, 798, 742, 727, 673 cm^−1^. ^1^H-NMR: δ 13.78 (1H, s, OH), 11.91 (1H, s, SO_2_NH), 9.01 (1H, s, CH=N), 8.51 (2H, d, *J* = 4.9 Hz, H4′′, H6′′), 8.09–8.04 (2H, m, H2′, H6′), 7.76 (1H, d, *J* = 2.5 Hz, H4), 7.73 (1H, d, *J* = 2.6 Hz, H6), 7.63–7.58 (2H, m, H3′, H5′), 7.06 (1H, t, *J* = 4.9 Hz, H5′′). ^13^C-NMR: δ 164.77, 158.51, 156.96, 155.68, 150.47, 139.09, 132.93, 131.07, 129.28, 122.58, 122.11, 121.90, 120.69, 115.91. Anal. Calcd. for C_17_H_12_Cl_2_N_4_O_3_S (423.27): C, 48.24; H, 2.86; N, 13.24. Found: C, 48.03; H, 2.74; N, 13.30.

*4-[(3-Bromo-5-chloro-2-hydroxybenzylidene)amino]-N-(pyrimidin-2-yl)benzenesulfonamide* (**2n**). Pale orange solid; yield 87%; m.p. 253–256 °C (decomp.). IR: 3587 (O-H), 3032, 2938, 2807, 2729, 1581 (-HC=N-), 1489, 1443, 1411, 1338, 1294, 1279, 1199, 1184, 1159, 1092, 947, 869, 844, 793, 742, 723, 709, 675 cm^−1^. ^1^H-NMR: δ 13.93 (1H, s, OH), 11.91 (1H, s, SO_2_NH), 8.99 (1H, s, CH=N), 8.51 (2H, d, *J* = 4.9 Hz, H4′′, H6′′), 8.09–8.04 (2H, m, H2′, H6′), 7.88 (1H, d, *J* = 2.5 Hz, H4), 7.77 (1H, d, *J* = 2.6 Hz, H6), 7.65–7.59 (2H, m, H3′, H5′), 7.06 (1H, t, *J* = 4.9 Hz, H5′′). ^13^C-NMR: δ 164.83, 158.53, 156.96, 156.67, 150.32, 139.10, 135.71, 131.80, 129.28, 122.97, 122.15, 120.41, 115.95, 111.33. Anal. Calcd. for C_17_H_12_BrClN_4_O_3_S (467.72): C, 43.66; H, 2.59; N, 11.98. Found: C, 44.00; H, 2.61; N, 12.24.

*4-[(5-Chloro-2-hydroxy-3-iodobenzylidene)amino]-N-(pyrimidin-2-yl)benzenesulfonamide* (**2o**). Orange solid; yield 92%; m.p. 265–268 °C (decomp.). IR: 3566 (O-H), 3041, 2731, 1580 (-HC=N-), 1493, 1439, 1409, 1340, 1290, 1264, 1182, 1153, 1090, 944, 868, 842, 797, 742, 701, 671 cm^−1^. ^1^H-NMR: δ 11.89 (1H, s, SO_2_NH), 8.93 (1H, s, CH=N), 8.51 (2H, d, *J* = 4.9 Hz, H4′′, H6′′), 8.10–8.03 (2H, m, H2′, H6′), 7.98 (1H, d, *J* = 2.5 Hz, H4), 7.77 (1H, d, *J* = 2.6 Hz, H6), 7.65–7.58 (2H, m, H3′, H5′), 7.06 (1H, t, *J* = 4.9 Hz, H5′′). ^13^C-NMR: δ 164.83, 159.14, 158.38, 156.96, 150.30, 141.35, 132.54, 129.96, 129.27, 123.42, 122.17, 119.24, 112.29, 87.44. Anal. Calcd. for C_17_H_12_ClIN_4_O_3_S (514.72): C, 39.67; H, 2.35; N, 10.89. Found: C, 39.89; H, 2.46; N, 10.68.

*4-[(2-Hydroxy-3,5-diiodobenzylidene)amino]-N-(pyrimidin-2-yl)benzenesulfonamide* (**2p**). Red solid; yield 95%; m.p. 266–267.5 °C (decomp.). IR: 3567 (O-H), 3040, 1577 (-HC=N-), 1489, 1456, 1435, 1410, 1339, 1281, 1181, 1152, 1089, 932, 861, 833, 801, 743, 714, 662 cm^−1^. ^1^H-NMR: δ 11.90 (1H, s, SO_2_NH), 8.91 (1H, s, CH=N), 8.51 (2H, d, *J* = 4.9 Hz, H4′′, H6′′), 8.17 (1H, d, *J* = 2.1 Hz, H4), 8.08–8.04 (2H, m, H2′, H6′), 7.99 (1H, d, *J* = 2.2 Hz, H6), 7.63–7.59 (2H, m, H3′, H5′), 7.06 (1H, t, *J* = 4.9 Hz, H5′′). ^13^C-NMR: δ 164.68, 159.98, 158.52, 156.96, 150.25, 149.04, 141.51, 138.99, 129.26, 122.15, 120.76, 115.91, 88.42, 81.67. Anal. Calcd. for C_17_H_12_I_2_N_4_O_3_S (606.18): C, 33.68; H, 2.00; N, 9.24. Found: C, 33.47; H, 2.14; N, 8.97.

*4-{[(5-Nitrofuran-2-yl)methylene]amino}-N-(pyrimidin-2-yl)benzenesulfonamide* (**2q**). Yellowish solid; yield 85%; m.p. 207–209 °C. IR: 3419, 2875, 1600, 1577 (-HC=N-), 1533, 1501, 1489, 1444, 1408, 1354, 1340, 1300, 1240, 1159, 1094, 1022, 970, 935, 831, 814, 803, 740, 722, 673, 659 cm^−1^. ^1^H-NMR: δ 11.42 (1H, s, SO_2_NH), 8.49 (2H, d, *J* = 4.9 Hz, H4′′, H6′′), 7.78–7.74 (2H, m, H2′, H6′), 7.73–7.69 (2H, m, CH=N, furan H4), 7.02 (1H, t, *J* = 4.9 Hz, H5′′), 6.95–6.90 (2H, m, H3′, H5′), 6.00 (1H, d, *J* = 8.5 Hz, furan H3). ^13^C-NMR: δ 158.46, 158.39, 157.28, 155.29, 151.45, 149.68, 129.65, 128.61, 115.84, 113.75, 112.83, 112.31. Anal. Calcd. for C_15_H_11_N_5_O_5_S (373.34): C, 48.26; H, 2.97; N, 18.76. Found: C, 48.20; H, 2.83; N, 18.61.

### 3.2. Antimicrobial Activity

#### 3.2.1. In Vitro Antibacterial Activity

The antibacterial activities were assayed against four Gram-positive and four Gram-negative strains: *Staphylococcus aureus* CCM 4516/08, methicillin-resistant *Staphylococcus aureus* H 5996/08 (MRSA), *Staphylococcus epidermidis* H 6966/08, *Enterococcus faecalis* J 14365/08; *Escherichia coli* CCM 4517, *Klebsiella pneumoniae* D 11750/08, extended-spectrum β-lactamase positive *Klebsiella pneumoniae* J 14368/08, and *Pseudomonas aeruginosa* CCM 1961. The microdilution broth method modified according to CLSI standard M07-A10 with Mueller-Hinton broth (HiMedia Laboratories, Mumbai, India) adjusted to pH 7.4 (±0.2) was used. The tested compounds were dissolved in DMSO to final concentrations ranging from 500 to 0.49 µM. Bacitracin (BAC) and sulfadiazine **1** were used as the reference drugs. A bacterial inoculum in sterile water was prepared to reach 0.5 according to McFarland scale (1.5 × 10^8^ CFU/mL). The minimum inhibitory concentrations were assayed as a reduction of growth compared to the control. The results were analysed visually. The MIC values were determined after 24 and 48 h of incubation in the dark at 35 °C (±0.1) in a humid atmosphere [16].

#### 3.2.2. In Vitro Antimycobacterial Activity

The sulfadiazine derivatives **2** were evaluated to determine their in vitro antimycobacterial activity against *Mycobacterium tuberculosis* 331/88 (H_37_Rv; the dilution of this strain was 10^−3^), *Mycobacterium avium* 330/88 (resistant to INH, rifamycins, ofloxacin, and ethambutol; dilution of 10^−5^), and two strains of *M. kansasii*, namely 235/80 (dilution of 10^−4^) and the clinically isolated strain 6509/96 (dilution of 10^−5^), using a previously described method [16]. The following concentrations were used: 1000, 500, 250, 125, 62.5, 32, 16, 8, 4, 2, and 1 μM. The MIC (reported in μM) is the lowest concentration at which the complete inhibition of mycobacterial growth was observed. The MICs were determined after incubation at 37 °C for 14 and 21 d, for *M. kansasii* additionally for 7 d. Isoniazid (INH) and the parent sulfadiazine **1** were chosen as the reference drugs. The inhibition of isocitrate lyase was determined according to [21].

#### 3.2.3. In Vitro Antifungal Activity

The antifungal properties were evaluated against four *Candida* strains (*Candida albicans* ATCC 44859, *Candida tropicalis* 156, *Candida krusei* E28, and *Candida glabrata* 20/I), *Trichosporon asahii* 1188 and three strains of filamentous fungi (*Aspergillus fumigatus* 231, *Lichtheimia corymbifera* 272, and *Trichophyton interdigitale* 445). The microdilution broth method with slight modifications was used according to the CLSI M27-A3 and M38-A2 guidelines in RPMI 1640 with glutamine (KlinLab, Prague, Czech Republic) buffered to pH 7.0 with 0.165 mol of 3-morpholino-propane-1-sulphonic acid (Sigma-Aldrich). DMSO served as a diluent for all the compounds. In yeast, the final size of the inoculum was 5 × 10^3^ ± 0.2 CFU/mL, and in the case of the moulds, the final size of the inoculum was 0.5–5 × 10^4^ CFU/mL. Fluconazole (FLU) was involved as the reference drug. The MIC values were assayed as inhibition of growth compared to the control. The results were analysed visually. The MIC values were determined after 24 and 48 h of incubation in the dark at 35 °C (±0.1) in a humid atmosphere. For *T. interdigitale*, the final MIC values were determined after 72 and 120 h of incubation [17].

### 3.3. Cytotoxicity Evaluation

All of the compounds were tested to determine their cytotoxicity in the human hepatocellular liver carcinoma cell line HepG2 (passages 5–6 and 3–8; ECACC, Salisbury, UK) using a standard colorimetric method that involves measuring the reduction of a tetrazolium salt (CellTiter(R) 96 AQueous One Solution Cell Proliferation Assay; Promega, Fichtburg, MA, USA).

The cells were cultured in Minimum Essentials Eagle Medium (Sigma-Aldrich) supplemented with 10% foetal bovine serum (PAA), 1% L-glutamine solution (Sigma-Aldrich) and a non-essential amino acid solution (Sigma-Aldrich) in a humidified atmosphere containing 5% CO_2_ at 37 °C. The investigated compounds were dissolved in DMSO and a small volume was added to the cell culture. The tested compounds were prepared in triplicates at concentrations ranging from 1 to 1500 µM. Simultaneously, the following types of controls were also included in triplicates: determination of 100% viability and 0% viability (the cells were treated with 10% DMSO), no cell control and vehiculum controls. After 24 h incubation in a humidified atmosphere containing 5% CO_2_ at 37 °C, the reagent from the kit CellTiter 96 was added. After 2 h incubation at 37 °C, absorbance of samples was recorded at 490 nm (Infinita M200, TECAN, Grödig, Austria). A standard toxicological parameter IC_50_ was calculated by nonlinear regression from a semilogarithmic plot of incubation concentration versus percentage of absorbance relative to untreated controls using GraphPad Prism 6 software (GraphPad Software Inc., San Diego, CA, USA). IC_50_ is the inhibitory concentration that reduces the cell viability to 50% of the maximal (control) viability.

## 4. Conclusions

In conclusion, we identified several interesting structure-activity relationships in some novel sulfadiazine Schiff bases. Our findings suggested that several were selective for microbial pathogens (*M. tuberculosis*, *M. kansasii*, *Staphylococcus* spp. including one MRSA strain, yeasts and *Trichophyton interdigitale*). 4-[(2-Hydroxybenzylidene)amino]-*N*-(pyrimidin-2-yl)benzenesulfonamide (**2a**) is active in the inhibition of tuberculous and atypical mycobacteria. The 2,5-dihydroxybenzaldehyde-derived Schiff base **2i** exhibited the highest selectivity for staphylococci and fungi. For these molecules, the desirable action is separated from an unwanted toxicity, thus constituting a promising hit for further structure optimization and development of potential antimicrobial agents. Further studies are required to gain a deeper understanding of their properties (especially mechanism of action and interactions with eukaryotic cells).

## Figures and Tables

**Table 1:** Antibacterial activity of sulfadiazine derivatives **2**.

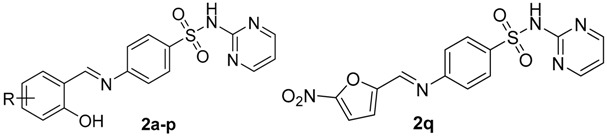																	
Code	R	MIC (µM)															
		SA		MRSA		SE		EF		EC		KP		KP-E		PA	
		24 h	48 h	24 h	48 h	24 h	48 h	24 h	48 h	24 h	48 h	24 h	48 h	24 h	48 h	24 h	48 h
**2a**	H	500	500	250	250	62.5	62.5	**31.25**	**62.5**	>500	>500	>500	>500	>500	>500	**500**	**500**
**2b**	5-F	>250	>250	>250	>250	>250	>250	>250	>250	>250	>250	>250	>250	>250	>250	>250	>250
**2c** [4]	5-Cl	250	250	125	125	>500	>500	>500	>500	>500	>500	>500	>500	>500	>500	>500	>500
**2d**	5-Br	500	500	500	500	250	250	>500	>500	500	500	500	500	500	500	**500**	500
**2e**	5-I	250	250	250	250	62.5	125	250	500	**250**	**250**	500	500	500	500	**500**	**500**
**2f**	5-NO_2_	250	250	250	250	500	500	500	500	**250**	**250**	>500	>500	500	500	**500**	**500**
**2g**	5-CH_3_	>250	>250	125	125	125	125	>250	>250	>250	>250	>250	>250	>250	>250	>250	>250
**2h**	5-CH_3_O	500	500	>500	>500	62.5	62.5	>500	>500	>500	>500	>500	>500	>500	>500	**500**	**500**
**2i**	5-OH	31.25	31.25	31.25	31.25	**15.62**	**15.62**	250	250	500	500	>500	>500	>500	>500	**500**	**500**
**2j**	5-*tert*-Bu	>125	>125	>125	>125	>125	>125	>125	>125	>125	>125	>125	>125	>125	>125	>125	>125
**2k**	6-Cl	>250	>250	>250	>250	>250	>250	>250	>250	>250	>250	>250	>250	>250	>250	>250	>250
**2l**	3-Cl	>500	>500	>500	>500	>500	>500	>500	>500	>500	>500	>500	>500	>500	>500	>500	>500
**2m**	3,5-Cl_2_	62.5	62.5	62.5	62.5	31.25	31.25	>500	>500	>500	>500	>500	>500	500	500	>500	>500
**2n**	3-Br-5-Cl	31.25	31.25	**15.62**	**15.62**	31.25	31.25	>500	>500	500	500	>500	>500	500	500	>500	>500
**2o**	3-I-5-Cl	**15.62**	**15.62**	**15.62**	**15.62**	31.25	31.25	250	250	**250**	**250**	500	500	500	500	**500**	**500**
**2p**	3,5-I_2_	**7.81**	**7.81**	**7.81**	**7.81**	**7.81**	**7.81**	125	125	**125**	**125**	**125**	**125**	**125**	**125**	**500**	**500**
**2q**	-	125	125	125	125	31.25	31.25	>500	>500	>500	>500	500	500	500	500	**250**	**250**
**SDZ 1**	-	500	>500	500	>500	>500	>500	>500	>500	>500	>500	>500	>500	>500	>500	>500	>500
**BAC**	-	7.81	15.62	15.62	15.62	15.62	31.25	15.62	62.5	>500	>500	>500	>500	>500	>500	>500	>500

BAC = bacitracin; SDZ = sulfadiazine. SA: *Staphylococcus aureus* CCM 4516/08; MRSA: methicillin-resistant *Staphylococcus aureus* H 5996/08; SE: *Staphylococcus epidermidis* H 6966/08; EF: *Enterococcus faecalis.* J 14365/08. *Escherichia coli* CCM 4517, *Klebsiella pneumoniae* D 11750/08; ESBL-positive *Klebsiella pneumoniae* J 14368/08; PA: *Pseudomonas aeruginosa* CCM 1961. One or two of the best MIC value(s) for each strain are shown in bold.

**Table 2:** Antimycobacterial activity of sulfadiazine derivatives **2**.

Code	R	MIC (µM)										
		*Mtb.* 331/88		*M. avium* 330/88		*M. kansasii* 235/80			*M. kansasii* 6509/96			Clog*P*
		14 d	21 d	14 d	21 d	7 d	14 d	21 d	7 d	14 d	21 d	
**2a**	H	**16**	**32**	**125**	**250**	**8**	**16**	32	**8**	**16**	32	2.5
**2b**	5-F	125	125	500	1000	62.5	125	250	125	250	250	2.65
**2c [4]**	5-Cl	125	250	**125**	**125**	**8**	**16**	32	**16**	32	32	3.05
**2d**	5-Br	**16**	**32**	**125**	**125**	32	62.5	62.5	32	32	62.5	3.32
**2e**	5-I	62.5	125	**125**	**250**	32	62.5	125	32	32	62.5	3.85
**2f**	5-NO_2_	250	250	500	1000	125	250	500	125	250	250	2.15
**2g**	5-CH_3_	125	125	500	1000	125	125	250	62.5	62.5	125	2.98
**2h**	5-CH_3_O	**32**	62.5	**125**	**125**	**16**	32	62.5	**16**	**16**	32	2.37
**2i**	5-OH	125	125	500	1000	62.5	125	125	62.5	125	125	2.11
**2j**	5-*tert*-Bu	**32**	62.5	**125**	**125**	**16**	32	62.5	**16**	**16**	32	4.2
**2k**	6-Cl	125	125	**250**	**250**	62.5	125	250	62.5	125	125	3.05
**2l**	3-Cl	**32**	62.5	**125**	**250**	**16**	32	62.5	**16**	**16**	32	3.05
**2m**	3,5-Cl_2_	125	125	**250**	500	125	250	500	62.5	125	250	3.61
**2n**	3-Br-5-Cl	125	125	**250**	500	125	125	250	62.5	125	125	3.88
**2o**	3-I-5-Cl	125	125	500	500	125	250	250	62.5	125	125	4.41
**2p**	3,5-I_2_	250	250	**250**	500	125	250	250	62.5	125	250	5.21
**2q**	-	125	125	500	500	125	250	250	62.5	62.5	125	ND
**SDZ 1**	-	32	62.5	62.5	62.5	16	16	32	8	8	8	0.21
**INH**	-	0.5	1	>250	>250	>250	>250	>250	8	8	8	-

INH = isoniazid; SDZ = sulfadiazine. *Mtb.* = *Mycobacterium tuberculosis*. ND = not determined. One or two of the best MIC value(s) for each strain are shown in bold.

**Table 3:** Antifungal activity of sulfadiazine derivatives **2**.

Code	R	MIC (µM)															
		CA		CT		CK		CG		TA		AF		LC		TI	
		24 h	48 h	24 h	48 h	24 h	48 h	24 h	48 h	24 h	48 h	24 h	48 h	24 h	48 h	72 h	120 h
**2a**	H	>500	>500	>500	>500	>500	>500	>500	>500	>500	>500	>500	>500	>500	>500	125	125
**2b**	5-F	>250	>250	>250	>250	>250	>250	>250	>250	>250	>250	>250	>250	>250	>250	>250	>250
**2c [4]**	5-Cl	125	125	>500	>500	>500	>500	125	125	62.5	125	>500	>500	>500	>500	>500	>500
**2d**	5-Br	125	125	250	250	250	250	62.5	62.5	62.5	62.5	250	250	>500	>500	62.5	62.5
**2e**	5-I	62.5	125	125	125	62.5	125	62.5	62.5	125	125	250	250	>500	>500	31.25	31.25
**2f**	5-NO_2_	500	500	>500	>500	>500	>500	250	250	>500	>500	>500	>500	500	500	62.5	62.5
**2g**	5-CH_3_	>500	>500	>500	>500	>500	>500	>500	>500	>500	>500	>500	>500	>500	>500	>500	>500
**2h**	5-CH_3_O	500	500	500	500	>500	>500	500	500	500	500	>500	>500	>500	>500	62.5	62.5
**2i**	5-OH	15.62	15.62	**15.62**	**15.62**	**31.25**	**31.25**	15.62	15.62	15.62	15.62	**125**	**125**	500	500	**1.95**	**1.95**
**2j**	5-*tert*-Bu	>125	>125	>125	>125	>125	>125	>125	>125	>125	>125	>125	>125	>125	>125	62.5	62.5
**2k**	6-Cl	>250	>250	>250	>250	>250	>250	>250	>250	>250	>250	>250	>250	>250	>250	>250	>250
**2l**	3-Cl	62.5	62.5	125	125	250	250	125	125	125	125	250	250	>500	>500	31.25	31.25
**2m**	3,5-Cl_2_	62.5	62.5	125	125	62.5	62.5	31.25	31.25	62.5	62.5	250	250	500	500	125	125
**2n**	3-Br-5-Cl	31.25	31.25	62.5	62.5	62.5	62.5	31.25	31.25	62.5	62.5	250	250	500	500	62.5	62.5
**2o**	3-I-5-Cl	**7.81**	**7.81**	**7.81**	**7.81**	**7.81**	**7.81**	**7.81**	**7.81**	**7.81**	**7.81**	**62.5**	**62.5**	250	250	**1.95**	**1.95**
**2p**	3,5-I_2_	**3.9**	**3.9**	**7.81**	**7.81**	**7.81**	**7.81**	**3.9**	**3.9**	**3.9**	**3.9**	**62.5**	**62.5**	**62.5**	**62.5**	**15.62**	**15.62**
**2q**	-	250	250	250	250	250	250	250	250	500	500	500	500	>500	>500	250	250
**FLU**	-	0.24	0.24	>500	>500	125	250	31.25	500	250	500	>500	>500	>500	>500	7.81	125

FLU = fluconazole; SDZ = sulfadiazine **1**. CA: *Candida albicans* ATCC 44859; CT: *Candida tropicalis*; CK: *Candida krusei* E28; CG: *Candida glabrata* 20/I; TA: *Trichosporon asahii* 1188; AF: *Aspergillus fumigatus* 231; LC: *Lichtheimia corymbifera* 272; TI: *Trichophyton interdigitale* 445. One or two of the best MIC value(s) for each strain are shown in bold.

**Table 4:** SAR for the antimycobacterial activity.

Strain	Improving Activity	Decreasing Activity
*M. tuberculosis*	H, 5-Br, 5-CH_3_O, 5-*t*-Bu, 3-Cl	5-Cl, 5-NO_2_, 3,5-I_2_
*M. avium*	H, 5-Cl, 5-Br, 5-I, 5-CH_3_O, 5-*t*-Bu, 3-Cl	5-F, 5-NO_2_, 5-CH_3_, 5-OH, 3-I-5-Cl, 5-NO_2_-furylidene
*M. kansasii*	H, 5-Cl, 5-Br, 5-CH_3_O, 5-*t*-Bu, 3-Cl	5-F, 5-NO_2_, 5-CH_3_, 3,5-X_2_, 3-X-5-Cl, 5-NO_2_-furylidene

**Table 5:** Cytotoxicity for HepG2 cells and selectivity.

Code	R	IC_50_ (µM)	Range of Concentrations Tested	SI for *Staphylococci* (MRSA/SE)	SI for *Candida* sp.	SI for TI	SI for *Mtb.*	SI for *M. kansasii*
**2a**	H	>500 *	1–500	>2/>8	inactive	>4	**>15.63**	**>15.63**
**2b**	5-F	>250 *	1–250	inactive	inactive	inactive	>2	>1
**2c**	5-Cl	NT	NT	NT	NT	inactive	NT	NT
**2d**	5-Br	159.1	1–500	0.32/0.64	0.64–2.55	2.55	4.98–9.94	2.55–4.97
**2e**	5-I	156.0	1–500	0.62/1.25–2.50	1.25–2.50	4.99	1.25–2.50	1.25–4.88
**2f**	5-NO_2_	140.5	1–500	0.56/0.28	≤0.56	2.25	0.56	0.28–1.12
**2g**	5-CH_3_	569.5 **	1–500	4.56	inactive	inactive	4.56	2.28–9.11
**2h**	5-CH_3_O	351.1	1–500	inactive/5.62	≤0.70	5.62	5.62–**11.24**	5.62–**21.94**
**2i**	5-OH	>500	1–500	**>16/>32**	**>16**	**>256.41**	>4	>4
**2j**	5-*tert*-Bu	45.6	1–500	inactive	inactive	0.73	0.73–1.46	0.73–2.85
**2k**	6-Cl	102.20	1–500	inactive	inactive	inactive	0.82	0.41–1.64
**2l**	3-Cl	160.5	1–500	inactive	0.64–2.57	5.14	2.57–5.14	2.57–**10.03**
**2m**	3,5-Cl_2_	114.30	1–500	1.83/3.66	0.91–3.66	0.91	0.91	0.23–1.83
**2n**	3-Br-5-Cl	92.94	1–500	5.95/2.98	1.49–2.97	1.49	0.74	0.37–1.49
**2o**	3-I-5-Cl	59.23	1–500	3.79/1.90	7.58	**30.37**	0.47	0.24–0.95
**2p**	3,5-I_2_	38.0	1–500	4.87	4.87–9.73	2.43	0.15	0.15–0.61
**2q**	-	16.3	1–500	0.13/0.52	0.07	0.07	0.13	0.07–0.26
**SDZ 1**	-	>1500	1–1500	>3 (24 h)	inactive	inactive	**>24**	**>46.88**

* Measurement at higher concentration was not possible due to the precipitation of the compound in the medium. ** an estimated value based on the curve. SI = IC_50_/MIC. SDZ: sulfadiazine. NT = not tested; MRSA: methicillin-resistant *Staphylococcus aureus*; SE: *Staphylococcus epidermidis*; TI: *Trichophyton interdigitale*. The SI values higher than 10 are shown in bold.
